# Identification and characterization of a nonpeptidic cyclophilin ligand with antiviral activity against feline and porcine α-coronaviruses

**DOI:** 10.1186/s13567-025-01654-1

**Published:** 2025-11-05

**Authors:** Manon Delaplace, Mantasha Khan, Hélène Huet, Laurent Softic, Nazim Ahnou, Lionel Bigault, Jean-François Guichou, Abdelhakim Ahmed-Belkacem, Quentin Nevers, Sophie Le Poder

**Affiliations:** 1 UMR 1161 Virologie, INRAE, Laboratoire de Santé Animale, VIROLOGIE, École Nationale Vétérinaire d’Alfort, Anses, 94700 Maisons-Alfort, France; 2 Unité de Virologie Et Immunologie Moléculaires (VIM), INRAE, Université Paris-Saclay, Jouy-en-Josas, France; 3 Viral Genetics and Biosecurity Unit (GVB), French Agency for Food, Environmental and Occupational Health Safety (Anses), Ploufragan, France; 4 Équipe “Virus, Hépatologie, Cancer”, INSERM U955, IMRB, Université Paris-Est, Créteil, France; 5 Centre de Biologie Structurale (CBS), CNRS, INSERM, Université de Montpellier, Montpellier, France; 6 Francis Crick Institute, London, UK

**Keywords:** Animal coronaviruses, FIPV, TGEV, PEDV, cyclophilins, antivirals

## Abstract

Coronaviruses (CoVs) are emerging pathogens that have been extensively studied over the last twenty years and can cause acute respiratory diseases in humans, as exemplified by the SARS-CoV-2 pandemic. CoVs are also known for their importance in veterinary medicine and are responsible for severe pathologies in pets and livestock. These include feline infectious peritonitis virus (FIPV), which causes fatal disease in cats. In livestock, porcine CoVs such as transmissible gastroenteritis virus (TGEV) and porcine epidemic diarrhoea virus (PEDV) are the causative agents of acute enteric disease in piglets, which has a high mortality rate and a significant impact on the pork industry. In addition, animal CoVs may represent zoonotic reservoirs. Therefore, efficient antiviral strategies are needed to inhibit the replication of CoVs that infect various animal species. Here, we synthesized twenty small-molecule ligands that target cyclophilins, a family of cellular chaperones hijacked by several viruses, including CoVs. We screened their antiviral activity against feline and porcine α-CoVs and identified F83233 as a potent inhibitor of FIPV, TGEV and PEDV replication at micromolar concentrations that was effective in feline, porcine, and simian cells. As cyclophilins are highly conserved among mammals, F83233 could be a promising antiviral to treat different animal and zoonotic CoVs.

## Introduction

Coronaviruses (CoVs) are enveloped viruses with a nonsegmented, single-stranded RNA genome of positive polarity. In the *Coronaviridae* family, the subfamily *Orthocoronavirinae* is composed of four genera: *Alphacoronavirus*, *Betacoronavirus*, *Gammacoronavirus* and *Deltacoronavirus*. α-CoVs and β-CoVs infect mainly mammals, whereas γ-CoVs and δ-CoVs infect primarily birds, although some members also infect mammals [1]. CoVs have a considerable impact on public health: since 2002, they have been recognized as emerging and important pathogens in humans. Indeed, three zoonotic CoVs belonging to the β-CoV genus that induce severe pulmonary diseases have emerged in humans: SARS-CoV in 2002, MERS-CoV in 2012 and SARS-CoV-2 in 2019 [2–4]. The emergence of these viruses, especially SARS-CoV-2, the etiological agent of COVID-19, has led to a significant increase in knowledge about β-CoV biology and pathophysiology.

In veterinary medicine, CoVs are well known to trigger the development of various diseases, sometimes exhibiting a complex pathophysiology with a tropism that is not restricted to the respiratory tract [5, 6]. The genus *Alphacoronavirus* contains an important number of pathogens that infect domestic and livestock animals, and effective treatments or vaccines are often lacking for these viruses. Among them, FIPV (feline infectious peritonitis virus) causes a fatal disease in cats, feline infectious peritonitis (FIP) [7]. FIP is considered one of the leading causes of death in communal cats, with a 100% lethality rate, although treatment with GS-441524 now constitutes an excellent therapeutic strategy [8, 9]. Feline CoVs (FCoVs) are classified into two biotypes: feline enteric CoV (FeCV) and feline infectious peritonitis virus (FIPV). FeCV is endemic in cats and avirulent, inducing mild or subclinical digestive symptoms, whereas FIP disease is caused by virulent FIPV strains [7]. While FeCV has strict intestinal tropism, FIPV is able to infect monocytes and macrophages [10], allowing the virus to spread in various organs and leading to systemic infection. In addition to biotypes, FCoV can also be subdivided, on the basis of serological responses, into two serotypes: FCoV-I, the source of most natural infections among cats [11], and FCoV-II, resulting from recombination between FCoV-I and canine CoV type II (CCoV-II) [12]. In contrast to FCoV-I, FCoV-II replicates readily in feline cell lines such as Crandell-Rees feline kidney (CRFK) cells.

Several α-CoVs also threaten farms and livestock. For example, six CoVs can infect pigs, among which four induce clinically indistinguishable severe digestive disease, with case‒fatality ratios of 80–100% in piglets that are less than 10 days old [13, 14]. Transmissible gastroenteritis virus (TGEV) is the prototype porcine CoV that shares important sequence homology with FIPV. Interestingly, TGEV also exhibits dual tropism in vivo for the intestinal and respiratory epithelia [5]. In the 1980s, a deletion in the n-terminal domain of the spike protein led to the global emergence of a TGEV variant called porcine respiratory CoV (PRCV), which has lost enteric tropism and virulence [5]. As antibodies elicited against PRCV protect against TGEV, the latter is now well controlled. Porcine epidemic diarrhoea virus (PEDV) emerged in the 1970s: ancestral PEDV strains have been contained by vaccines, but PEDV has reemerged since the 2010s with the presence of new strains that render vaccines less effective [15–17]. The re-emergence of PEDV in the United States of America in 2013 led to the loss of 10% of pig herds, with a considerable economic impact [18, 19]. These PEDV strains now circulate globally [20]. Other porcine CoVs, such as swine acute diarrhoea syndrome CoV (SADS-CoV) and porcine Deltacoronavirus (PDCoV), are emerging viruses with documented zoonotic potential [21, 22]. Considering the impressive capacity of CoVs to overcome the species barrier [23] and the regular and severe resulting epidemics, it is of utmost importance to find not only treatment for a specific pathogenic animal CoV but also antiviral strategies that could be applied to a broad spectrum of CoVs, therefore preventing future emerging viruses.

CoVs rely on various host cell proteins to complete their life cycle, from entry to replication and assembly [24–29]. One important family of host proteins involved is cyclophilins, which are conserved cellular proteins present in both prokaryotes and eukaryotes [30]. Cyclophilins share a common peptidyl-prolyl cis/trans isomerase domain (PPIase) [30], which catalyzes the interconversion of the proline configuration [31]. Cyclophilins are known to play critical roles in the replication of different viruses [32], including DNA viruses [33] and negative- and positive-sense RNA viruses [34, 35]. Although their precise role and mechanism of action are unclear, it is suggested that they impact CoV replication [36]. Cyclosporin A (CsA), a macrocyclic inhibitor of cyclophilins, and its nonimmunosuppressive derivatives, such as alisporivir (ALV), inhibit CoV replication in different genera [37–39]. Gene knockout or siRNA-mediated silencing of cyclophilins has been shown to render cells nonpermissive or significantly reduce the replication of several CoVs in vitro [40, 41]. Cyclophilins have also been identified as interactors of several viral proteins of CoVs, including those of SARS-CoV [42] and HCoV-229E [43]. Therefore, cyclophilins appear to be a target of choice for the development of antiviral compounds capable of blocking the replication of several coronaviruses. In addition, targeting host factors that support viral replication, such as cyclophilins, makes it more difficult for viral escape variants to emerge [44].

We present here the characterization of the antiviral effects of nonpeptidic, nonimmunosuppressive small-molecule cyclophilin inhibitors [45] (“SMCypI”) against a feline CoV of major veterinary interest, FIPV, and two enteric porcine CoVs, TGEV and PEDV. Following the synthesis and screening of twenty SMCypI, we identified a compound referred to as F83233 as a potent anti-FIPV compound in feline cells. This molecule also potently inhibited the replication of TGEV and PEDV in porcine and simian cells, respectively, demonstrating its potential for the development of optimized antivirals effective in treating CoV diseases in animals and ultimately preventing the zoonotic risk caused by these pathogens.

## Materials and methods

### Cells and virus strains

Crandell-Rees feline kidney (CRFK), swine testicular (ST), porcine kidney (PK15) and Vero cells were cultivated at 37 °C with 5% CO_2_ in Dulbecco’s modified Eagle’s medium (DMEM, Gibco) supplemented with 10% foetal bovine serum, 1% penicillin‒streptomycin, 1% sodium pyruvate and 1% nonessential amino acids. All the cells were passaged fewer than ten times for the infection experiments.

The serotype II FIPV 79-1146 was amplified from feline CRFK cells. TGEV (Purdue strain) was amplified from porcine ST cells. PEDV (CV777 strain) was amplified from simian Vero cells in the absence of serum and in the presence of 10 µg/mL trypsin (Thermo Fischer Scientific, Waltham, USA). The supernatants were harvested and ultracentrifuged. Infectious titres were determined via end-point dilution assays.

### PPIase enzyme assay

Human cyclophilin A was purified, and its PPIase activity in the absence or presence of SMCypI was measured as previously described [45]. Briefly, cyclophilin A PPIase activity was measured at 20 °C using the standard chymotrypsin-coupled assay. The assay buffer (25 mM HEPES and 100 mM NaCl, pH 7.8) and purified cyclophilin A were precooled to 4 °C. Then, 5 µL of 50 mg/mL chymotrypsin in 1 mM HCl was added (final reaction volume of 500 µL). The reaction was initiated by adding the peptide substrate Suc-Ala-Ala-Cis-Pro-Phe-pNA to a LiCl/TFE solution with rapid inversion. The absorbance of p-nitroaniline was monitored at 390 nm until the reaction was complete, i.e., when the absorbance reached a plateau (1 min). The final concentration of LiCl in the assay was 20 mM, and TFE was present at a concentration of 4% (v/v). The absorbance was measured every second using a spectrophotometer (BioMate3, Thermo Fischer Scientific). For the inhibition assessment, 5 µL of the tested compound in dimethyl sulfoxide (DMSO) was added to the cyclophilin A solution in the assay buffer. CsA (Sigma‒Aldrich, Saint-Louis, USA) was used as a positive control for PPIase inhibition in all measurements. IC_50_ values were calculated from dose‒response curves and reported as the mean ± standard deviation of two or more experiments.

### SMCypI screening for FIPV infection

CRFK cells were plated in a 96-well plate at a density of 2 × 10^4^ cells/well and incubated for 24 h. The cells were infected for 1 h with FIPV at a multiplicity of infection (MOI) of 1 in the presence of 50 µM or 10 µM SMCypI or CsA, respectively. The inoculum was removed after 1 h, and inhibitors (50 μM SMCypI or 10 μM CsA) were added for 24 supplemental hours. The supernatants were collected and stored at −80 °C prior to the end-point dilution assays.

### FIPV titration by end-point dilution assay

CRFK cells were plated in a 96-well plate at a density of 2 × 10^4^ cells/well and incubated for 24 h. Serial tenfold dilutions (10^–1^ to 10^–6^) of viral supernatants, obtained with or without cyclophilin inhibitors, were added to CRFK cells in eight replicate wells per dilution. The viral inoculum was removed 2 h after infection, and the cells were incubated for 24 h at 37 °C. The cells were fixed via sequential incubation in absolute ethanol followed by 70% ethanol (10 min each at room temperature). FIPV antigens were then detected by immunofluorescence using ascitic fluid from an infected cat (dilution 1/500), followed by incubation with an anti-cat AlexaFluor-488-conjugated secondary antibody (Jackson ImmunoResearch, West Grove, USA). Wells containing fluorescent cells were counted. The 50% infectious dose (ID_50_) was calculated using the Spearman–Karber method. The results are presented as the means of ≥ 2 experiments performed in triplicate.

### Cytotoxicity assay

The cells were plated in a 96-well plate at a density of 2 × 10^4^ cells/well and incubated for 24 h. The culture medium was replaced with serial dilutions of culture medium containing three replicates of different concentrations (ranging from 0.9 μM to 25 μM) of F83233, F832 and F833 molecules, DMSO or media alone as controls. The plates were incubated for 24 h at 37 °C. Cytotoxicity was evaluated with the CellTiter-Glo Cell Viability Assay Kit (Promega, Madison, USA).

### Dose‒responses of SMCypI in CRFK, PK15 and Vero cells

CRFK, PK15 and Vero cells were plated in a 96-well plate at a density of 2 × 10^4^ cells/well and incubated for 18 h at 37 °C. The cells were infected with FIPV (MOI of 1), TGEV (MOI of 0.5) or PEDV (MOI of 0.5). Viral inoculation was performed in the presence of F83233, F832 and F833 at concentrations ranging from 0.1 μM to 25 μM for FIPV assays and from 0.78 µM to 12.5 µM for TGEV and PEDV. Two hours after viral inoculation, fresh medium supplemented with the same concentrations of inhibitors was added. The antiviral effect was measured at the peak of viral replication, i.e., after 24 h for FIPV and PEDV infection and after 48 h for TGEV infection. The experiments were repeated twice, and each condition was tested in duplicate or triplicate in independent wells.

### Time-of-drug addition assay

CRFK cells were plated in a 96-well plate at a density of 2 × 10^4^ cells per well and incubated for 24 h. The cells were infected with FIPV at an MOI of 1 in the presence of 5 µM F83233 (for 0 h post-infection (pi)) or in the presence of DMSO (for the other time points). After viral inoculation, the culture medium was removed, fresh medium containing 5 μM F83233 was added at 1, 3, 6, 9 or 18 h post-infection, and infection was stopped at 24 h post-inoculation. The cell supernatants were stored at −80 °C prior to end-point dilution to measure the effect of F83233 on FIPV infectivity under different conditions. The experiment was repeated three times, and each condition was tested in triplicate.

### Detection of TGEV and PEDV viral antigens by immunofluorescence

Fixed PK15 and Vero cells were processed similarly. TGEV infection was detected with a homemade anti-spike antibody (called 51.13 [46, 47], dilution 1/10 000) from a mouse and with an anti-mouse AlexaFluor-555-conjugated secondary antibody. PEDV infection was detected with polyclonal antibodies from pigs (a gift from Drs. Y. Blanchard and M. Contrant, Anses, Ploufragan, dilution 1/300) and with an anti-pig AlexaFluor-488-conjugated secondary antibody (SouthernBiotech, Birmingham, USA). Active PEDV replication was revealed with a mouse antibody directed against double-stranded RNA as recently described[48] (J2 from Cell Signaling Technology, Danvers, USA, dilution 1/1000), followed by incubation with an anti-mouse AlexaFluor-555-conjugated secondary antibody. The cells were counterstained with DAPI (Sigma‒Aldrich, final concentration of 1 µg/mL). Secondary antibodies (with the exception of the anti-pig at a 1/400 dilution) were used at a 1/800 dilution and were obtained from Molecular Probes (Thermo Fischer Scientific).

### Quantification of TGEV- and PEDV-infected areas and nuclei/syncytium

For each concentration of F832, F833 or F83233, 3 to 5 pictures were randomly taken with an epifluorescence microscope (Zeiss, Oberkochen, Germany) with a 10X objective. The area of staining was measured with ImageJ. The number of nuclei/syncytia (defined as PEDV-positive cells with > 4 nuclei) in PEDV-infected cells was manually counted using ImageJ. Blind analysis was performed to avoid bias.

### Alignment of amino acid sequences of cyclophilin A from different mammals

The cyclophilin sequences from humans (P62937), pigs (P62936), monkeys (P62938) and cats (Q8HXS3) were recovered from UniProt, aligned with ClustalW and processed with ESPript 3.

### In silico modelling and docking

The search for ligand‒cyclophilin 3D crystal complexes was performed using the @TOME‐3 server [49]. Ligand files were generated with MarvinSketch 6.2.2 for SMILES and the Grade server for mol2. Docking simulation of F83233 in complex with pig cyclophilin A was performed using the @TOME‐3 server with an anchor of PDB 4J5C. The images were generated using PyMOL and MarvinSketch.

## Results

### Screening of non-peptidic small-molecule cyclophilin inhibitors (SMCypI) on FIPV infection

**Figure 1:**
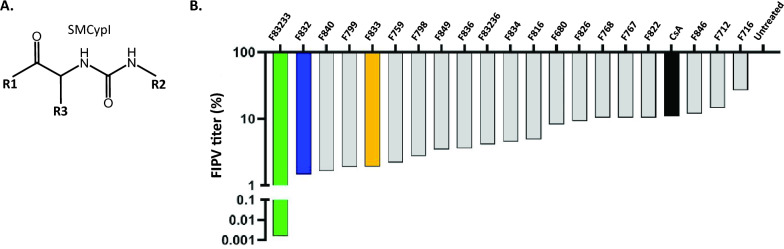
**Screening of the effects of nonpeptidic SMCypI on FIPV infection. A** SMCypI are composed of a common backbone with various substitutions that can be added at the R1, R2 and R3 regions, allowing the generation of a library with an important number of compounds. **B** SMCypI are more effective than CsA. The FIPV viral titre was measured 24 h after treatment with 20 different SMCypI (50 µM). CsA (black) was used at a final concentration of 10 µM. The results are normalized to those of untreated infected cells.

We previously developed SMCypI to obtain broad-spectrum antiviral agents [45] and showed that our first SMCypI series exhibited potent antiviral activity against hepatitis C virus replication in vitro [50] but that it was only modest in reducing the replication of HCoV-229E, with EC_50_ values ranging from 7 to 71 µM [45]. SMCypI have a common backbone in which various chemical moieties can be added at three distinct sites, referred to as R1, R2 and R3 (Figure 1A and Table 1), thus allowing the generation of new molecules (Table 1). We thus aimed to find more potent anti-CoV compounds in our enriched library.

**Table 1:**
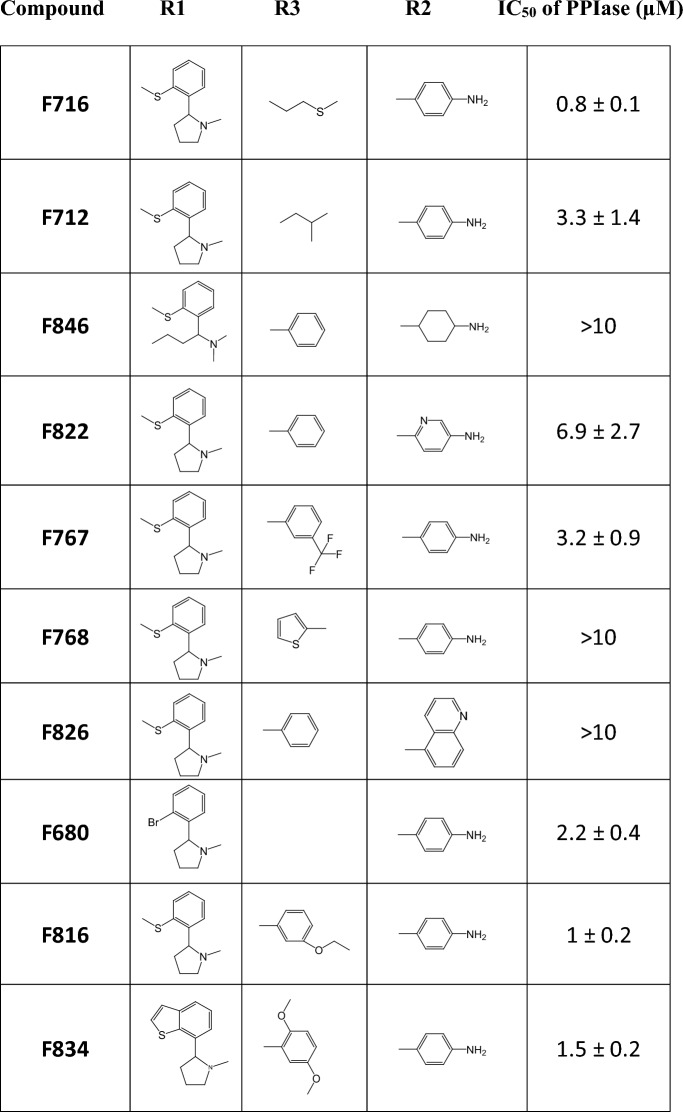

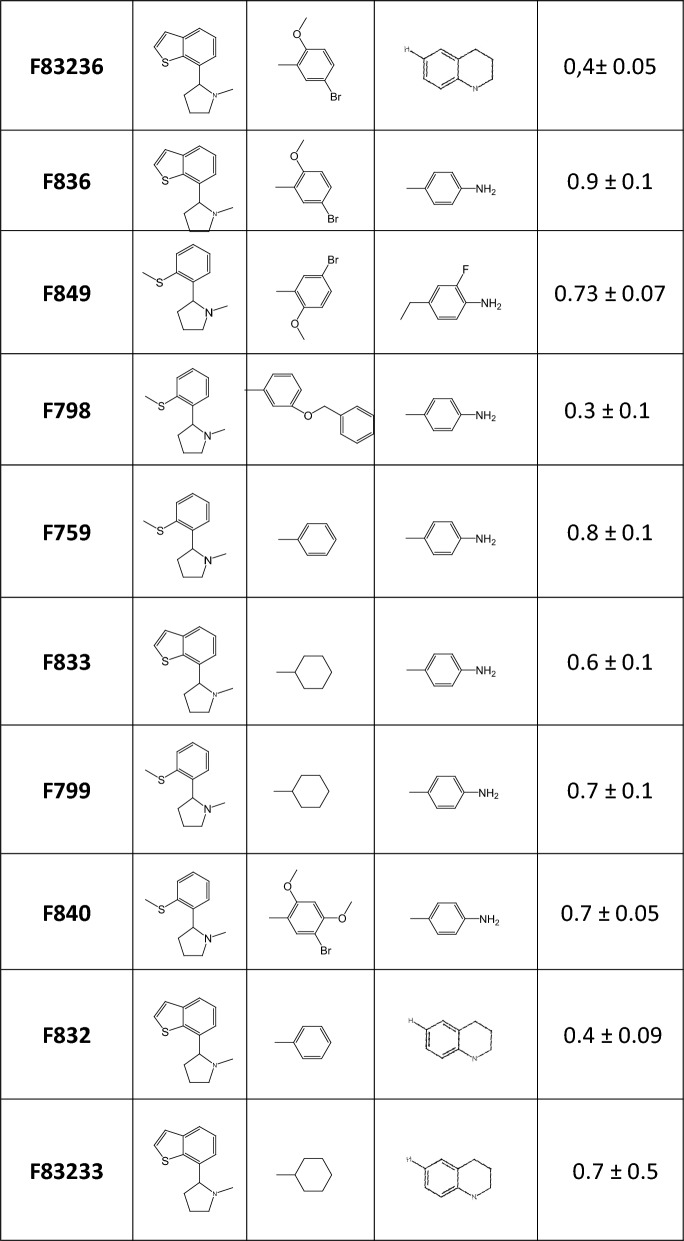
**Structures and anti-PPIase activities of SMCypI**.

We screened twenty different SMCypI (Figure 1B and Table 1) for their ability to reduce FIPV titre (strain 79–1146) in feline CRFK cells at a final concentration of 50 µM. We also used 10 µM Csa (black bar), a peptidic cyclophilin inhibitor with reported anti-CoV activity, including FIPV [37]. We observed that all the tested molecules modestly decreased FIPV infectious titre, with the exception of the F83233 compound (green bar), which inhibited the FIPV titre by more than 4 Log_10_ (Figure 1B).

### The increased anti-FIPV effect of F83233 compared with F832 and F833 is unrelated to the inhibition of cyclophilin A enzymatic activity

**Figure 2:**
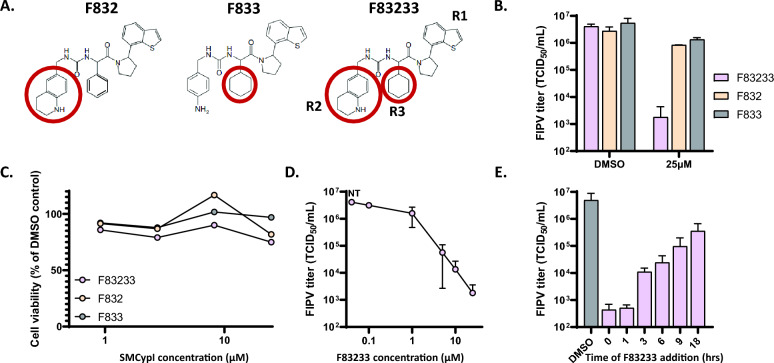
**Characterization of the anti-FIPV activity of SMCypI F83233. A** Chemical structures of the F832, F833 and F83233 molecules. F83233 harbours, at the R2 and R3 positions, chemical moieties from F832 and F833, respectively (red circles). **B** The antiviral effects of 25 µM F83233 compared with those of F832 and F833 were assessed by measuring the FIPV viral titre in the cell supernatants by TCID_50_ after treatment for 24 h with the drugs or DMSO. *n* = 2 independent experiments. **C** Cytotoxicity assays. Measurement of ATP release after treatment with increasing concentrations of F83233, F832 and F833. **D** Dose responses. FIPV viral titres were measured in cell supernatants after treatment with increasing concentrations of F83233. *n* = 2 independent experiments. **E** Time of addition assays. F83233 (5 µM) was added to the cells at various times post-infection (between 0 and 18 h), and FIPV titres from the cell supernatants were measured under different conditions. *n* = 3 independent experiments. N.T: not treated.

Interestingly, SMCypI F83233 is a “mix” of two compounds with apparent low anti-FIPV effects, F832 and F833 (blue and orange bars in Figure 1B, respectively), as it contains the same chemical moieties as these SMCypI at the R2 and R3 positions, respectively (Figure 2A and Table 1). We first confirmed the results from our screen showing a better anti-FIPV effect of F83233 than F832/F833 by measuring the virus titre in cell supernatants after FIPV infection in the presence of 25 µM of these three SMCypI. While F832 and F833 only modestly inhibited FIPV infectivity by ≈ 0.5 Log_10_ (Figure 2B), F83233 decreased the virus titre at this concentration by ≈ 3 Log_10_. We measured the ability of these three compounds to block the enzymatic activity of human cyclophilin A in an in vitro assay to assess whether this increased antiviral potency of F83233 was linked to an improved blockade of cyclophilin function (Table 1). However, we observed no significant differences, with IC_50_ values of 0.7 ± 0.5 µM for F83233, 0.4 ± 0.09 µM for F832 and 0.6 ± 0.1 µM for F833 (Table 1). Given that the apparent antiviral effect could sometimes be due to cellular toxicity, we treated CRFK cells for 24 h with increasing concentrations of the three compounds and observed a low cytotoxicity of the drugs up to 25 µM, which was not different between F83233 and F832/F833 (Figure 2C), an observation that was similar to that reported in the simian Vero cell line (Softic et al. submitted). Overall, the increased activity of F83233 compared with that of its “parental” molecules F832 and F833 is not due to improved anti-PPIase activity toward cyclophilin A or to cellular toxicity under our experimental conditions.

### Characterization of the anti-FIPV activity of F83233

To characterize the anti-FIPV activity of F83233, we first quantified FIPV infectious titres in the presence of increasing doses of SMCypI (Figure 2D). We demonstrated strong inhibition of FIPV infectivity, with a reduction in the viral titre of approximately 2 Log_10_ at 5 µM. Furthermore, the maximal inhibitory effect of F83233 was a decrease of approximately 3–4 Log_10_ at 12.5 µM. To better understand the mechanism of action of F83233 on the FIPV replication cycle, we added 5 µM F83233 at different times post-infection (0, 1, 3, 6, 9 and 18 h) and measured the infectious titres of the supernatants produced under different conditions (Figure 2E). The maximal antiviral effect of F83233 was observed when the molecule was added simultaneously with the virus or 1 h later. Its antiviral efficiency decreased by more than 1 Log_10_ between 1 and 3 h post-infection. From 3 to 18 h post-infection, F83233 continuously lost antiviral efficiency but was still effective 18 h post-infection, with ≈ 1 Log_10_ of inhibition compared with the DMSO control. Taken together, these data indicate that F83233 mainly blocks an early step of the FIPV replication cycle.

### Inhibition of TGEV infection in porcine cells by F83233

**Figure 3:**
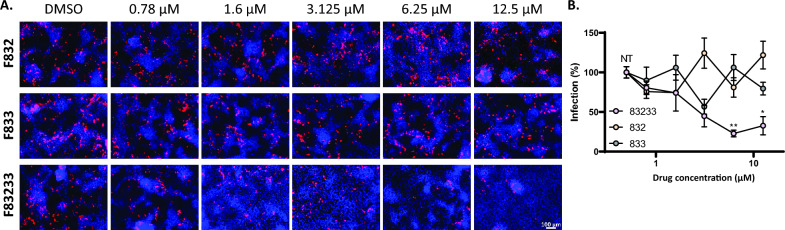
**Antiviral activity of SMCypI against TGEV in porcine cells. A** PK15 cells were infected with TGEV (Purdue strain) at an MOI of 0.5 for 48 h in the presence of increasing concentrations of SMCypI or only DMSO. Viral infection was detected by immunofluorescence using an anti-spike monoclonal antibody and an anti-mouse A-555 secondary antibody (red). The cell nuclei were counterstained with DAPI. Objective: 10X. **B** Quantification of TGEV inhibition by SMCypI. A total of 4–5 images were captured for each condition, with at least two images per well. The area of infection was then quantified with ImageJ. A figure from one experiment is representative of *n* = 2 independent experiments with 2 independent wells. N.T.: not treated. *p < 0.05, **p < 0.01 (one-way ANOVA followed by the Kruskal‒Wallis test).

We next evaluated whether F83233 could also inhibit infections caused by other α-CoVs of veterinary interest. We first studied infection by porcine TGEV (Purdue strain) in porcine kidney PK15 cells (Figures 3A and B). As for FIPV, we compared the antiviral potencies of F83233 and F832/F833. While F832 and F833 had no significant effect on TGEV infection even at 12.5 µM, F83233 was active at a concentration of 1.6 µM and was associated with an almost complete disappearance of infected cells at 6.25 µM and 12.5 µM (Figures 3A and B).

### Inhibition of PEDV infection in simian cells by F83233

**Figure 4:**
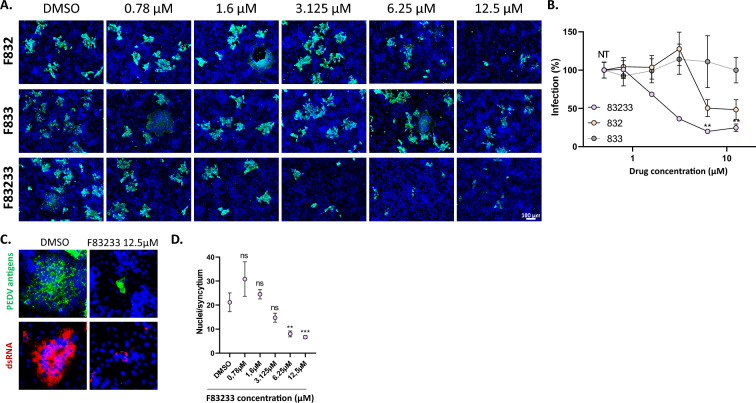
**Antiviral activity of SMCypI against PEDV in simian cells. A** Vero cells were infected with PEDV (CV777 strain) at an MOI of 0.5 for 24 h in the presence of increasing concentrations of SMCypI or only DMSO. Viral infection was detected by immunofluorescence using anti-PEDV porcine polyclonal antibodies and an anti-pig secondary antibody (green). The cell nuclei were counterstained with DAPI. Objective: 10X. **B** Quantification of PEDV inhibition by SMCypI. A total of 4–5 images were captured for each condition, with at least two images per well. The area of infection was then quantified with ImageJ. A figure from one experiment is representative of *n* = 2 independent experiments with 2 independent wells. **p < 0.01 (one-way ANOVA followed by the Kruskal‒Wallis test). NT: not treated. **C** SMCypI treatment drastically reduced syncytium formation. Infected Vero cells were treated with 12.5 µM F83233 and stained after fixation with anti-PEDV polyclonal antibodies and an anti-pig secondary antibody (green) or with a mouse antibody that recognizes double-strand RNA structures and an anti-mouse secondary antibody (red). Objective: 10X. **D** Quantification of the number of nuclei per syncytia after treatment with increasing doses of F83233. The number of nuclei per syncytium was counted in four different fields of cells (2 pictures per well). A figure from one experiment is representative of *n* = 2 independent experiments with 2 independent wells. ns: not significant. ***p < 0.001 (one-way ANOVA followed by the Kruskal‒Wallis test).

To study the broad-spectrum potential of F83233, we tested its antiviral effect on the replication of PEDV (CV777 strain), which is more genetically distant from FIPV. Similarly, in Vero cells infected with PEDV, we observed modest antiviral potency of F832 and F833, whereas F83233 caused an important reduction in infection at 3.125 µM and a maximal antiviral effect between 3.125 and 6.25 µM (Figures 4A and B).

In cultured cells, several CoVs are characterized by an atypical cytopathic effect, i.e., the formation of multinucleated cells referred to as syncytia, as a result of massive cell‒cell fusion triggered by the viral spike protein [51–53]. In Vero cells, PEDV appears to be one of the most fusogenic CoVs, and we observed several syncytia containing up to 100 nuclei under our experimental conditions (Figure 4C, DMSO condition). Using an antibody that recognizes double-stranded RNA structures [48] (which are formed during the replication of viruses with RNA genomes of positive polarity), we observed that syncytia are active sites of RNA replication, with dsRNA spots located around the packed nuclei (Figure 4C). Using 12.5 µM F83233, we noticed that SMCypI drastically decreased the size of the PEDV-induced syncytia and the number of nuclei per syncytium (Figures 4C and D) and markedly impacted the double-strand RNA staining corresponding to viral genome replication in the syncytia (Figure 4C).

## Discussion

The regular emergence of new CoVs necessitates the urgent development of antiviral tools with a broad pan-CoV spectrum. Two main antiviral methodologies are commonly employed: either to specifically target viral proteins or to target the cellular proteins that are necessary for viral replication. While the initial approach is more frequently used, it is not without its drawbacks. First, it is frequently virus specific, and second, it is difficult to extrapolate its antiviral efficiency to other CoVs. Finally, concerns about the emergence of antiviral-resistant mutants have been raised. Recently, a nucleoside analogue, GS-441524, a metabolite of the antiviral prodrug remdesivir, has been employed to treat FIP disease, with considerable success [8, 9]. However, this strategy may not be efficacious against SARS-CoV-2, for example [54]. In this study, we chose to target the cellular cyclophilin A (encoded by the PPIA gene), the most abundant cyclophilin, which has been shown to be necessary for the replication of multiple CoVs from different genera [32]. Furthermore, evidence has demonstrated that CsA, a macrocyclic peptide composed of 11 amino acids, forms a complex with cyclophilin A, thereby exerting an antiviral effect on at least FIPV, TGEV and PEDV [37, 41, 55]. Nevertheless, the immunosuppressive effect of this compound prevents its utilization as an antiviral therapeutic agent.

We previously developed low-molecular-weight, nonimmunosuppressive cyclophilin-inhibiting molecules (SMCypI). The anti-HCoV-229E activity of SMCypI was subsequently characterized, and it exhibited a modest antiviral effect [45]. However, the relatively simple chemistry of these molecules allows us to envisage numerous substitutions on their common backbone to generate a more diverse library (Figure 1A and Table 1). With this approach, we described a new antiviral molecule, F83233, with more efficient antiviral activity (approximately 3 Log_10_ viral reduction at 10 µM) than CsA against FIPV (Figures 1B and 2D). The potent antiviral activity of F83233 results directly from the easy possibility of “mixing” groups of two other compounds, such as F832 and F833. These data pave the way for the screening of more SMCypI and could lead to structure‒activity relationship studies for the design of molecules that are even more effective than F83233 against CoV (as well as other virus) replication.

For some viruses whose replication is cyclophilin dependent, it has been demonstrated that PPIase enzymatic activity is necessary [32]. For example, genome replication of the hepatitis C virus (HCV) absolutely requires the PPIase activity of cyclophilin A [32, 56]. Similarly, we previously reported a very good correlation between the ability of SMCypI to inhibit the PPIase activity of human cyclophilin A and its anti-HCV potency [50]. In our present study, however, the massive increase in anti-CoV activity of F83233 compared with F832, F833 or other molecules with submicromolar anti-PPIase IC_50_ values but low antiviral potency (F840, F799, F759) is not linked to the improved potency of F83233 on the PPIase activity of cyclophilin A (Figure 1B and Table 1). Similarly, CsA at 10 µM was less effective than F83233, whereas its IC_50_ for PPIase activity was 2 Log_10_ lower than that of F83233. Thus, the antiviral effect of SMCypI on CoV replication may be more complex than that observed in HCV, and these molecules could be very useful for better understanding the molecular mechanisms by which cyclophilin A and potentially other cyclophilins support CoV replication. Therefore, although we characterized the potent anti-CoV activity of F83233, we do not yet know the molecular and cellular basis of its antiviral activity against α-CoVs. Thus, further studies are needed to understand its precise mechanism of action.

**Figure 5:**
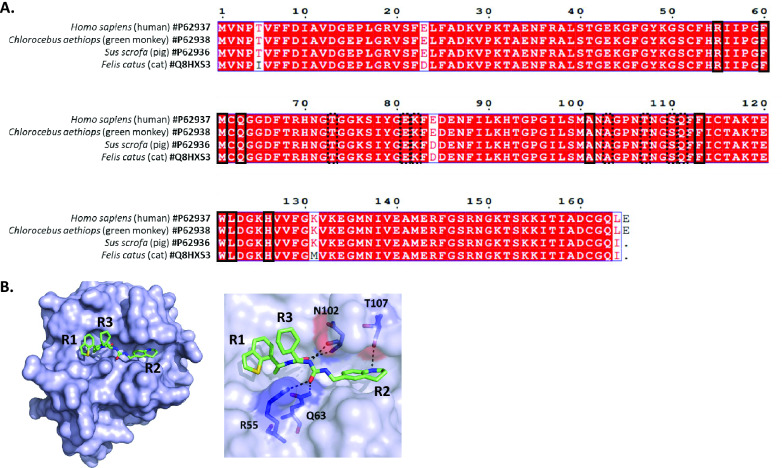
**High degree of conservation of cyclophilin A across mammals. A** Sequences of cyclophilin A (PPIA gene) were obtained from UniProt, aligned with ClustalW and processed with ESPript 3. The alignment shows a high degree of conservation of cyclophilin A from humans, monkeys, pigs and cats. Amino acids of the PPIase active site are in dashed rectangles, whereas amino acids that define the S2 “gatekeeper” pocket (which regulates substrate specificity [31]) are in dotted rectangles. **B** In silico modelling and docking. The cyclophilin A sequences from humans (P62937), pigs (P62936), monkeys (P62938) and cats (Q8HXS3) were recovered from UniProt. The ligand (F83233, in green)-cyclophilin 3D crystal complex was generated via the @TOME‐3 server [49]. Ligand files were generated with MarvinSketch 6.2.2 for SMILES and the Grade server for mol2. Docking simulation was performed via the @TOME‐3 server with an anchor of PDB 4J5C. The images were generated via PyMOL and MarvinSketch. Pig cyclophilin A is shown in the figure.

The development of broad-spectrum molecules can help prevent future panzootic events or even pandemics [57]. While specific antivirals or vaccines are being developed, this can, for example, provide a first line of defence in the event of viral emergence. Antivirals that target host factors may thus act on a wide range of viruses and animal species because of their conservation. In this study, the F83233 compound demonstrated robust antiviral efficacy against both FIPV and TGEV, which are genetically related. In addition, compared with TGEV, PEDV, a distinct α-CoV that exhibited only 31% and 29% amino acid homology with the spike and N proteins, respectively, was inhibited. The broad activity of F83233 on PEDV, FIPV and TGEV in simian, feline and porcine cells, respectively, demonstrates that SMCypI is an interesting lead for considering treatments that can be generalized to several animal species and represents a potential avenue to explore their ability to prevent the zoonotic risk posed by mammalian CoVs. This broad activity is consistent with the very strong conservation of cyclophilin A and its active PPIase site across mammals (Figure 5A). The 3D reconstruction of the complex formed by F83233 and cyclophilin A revealed identical interactions with cyclophilin A across the different species (human, porcine [shown in Figure 5B]), simian and feline). This paves the way for the study of the antiviral activity of SMCypI on various animal CoVs that infect more genetically distant species.

Although we identified a host-targeted compound capable of effectively inhibiting the replication of animal CoVs infecting different species, our study has several limitations. One of them is that the antiviral effect of F83233 was validated on laboratory-adapted viral strains in immortalized cell lines. When possible, it will be important to validate the effect of this SMCypI in more relevant cellular models (primary cells, organoids) with circulating field strains that may differ significantly from laboratory-adapted viral strains in terms of viral fitness, replication kinetics, and drug susceptibility. Finally, to determine whether this compound is a credible avenue for treating viral diseases in animals and the zoonotic risk posed by these viruses, in vivo studies should be carried out to investigate the pharmacokinetic properties of F83233 and to ultimately validate its antiviral effect in animals, such as pigs.

## Data Availability

The datasets used during and/or analysed during the current study are available from the corresponding author upon reasonable request.
